# Pre-operative gastrocnemius lengths in gait predict outcomes following gastrocnemius lengthening surgery in children with cerebral palsy

**DOI:** 10.1371/journal.pone.0233706

**Published:** 2020-06-05

**Authors:** Apoorva Rajagopal, Łukasz Kidziński, Alec S. McGlaughlin, Jennifer L. Hicks, Scott L. Delp, Michael H. Schwartz

**Affiliations:** 1 Department of Mechanical Engineering, Stanford University, Stanford, CA, United States of America; 2 Department of Bioengineering, Stanford University, Stanford, CA, United States of America; 3 Center for Gait and Motion Analysis, Gillette Children’s Specialty Healthcare, St. Paul, MN, United States of America; 4 Department of Orthopedic Surgery, University of Minnesota, Minneapolis, MN, United States of America; Virginia Tech, UNITED STATES

## Abstract

Equinus deformity is one of the most common gait deformities in children with cerebral palsy. We examined whether estimates of gastrocnemius length in gait could identify limbs likely to have short-term and long-term improvements in ankle kinematics following gastrocnemius lengthening surgery to correct equinus. We retrospectively analyzed data of 891 limbs that underwent a single-event multi-level surgery (SEMLS), and categorized outcomes based on the normalcy of ankle kinematics. Limbs with short gastrocnemius lengths that received a gastrocnemius lengthening surgery as part of a SEMLS (*case* limbs) were 2.2 times more likely than *overtreated* limbs (i.e., limbs who did not have short lengths, but still received a lengthening surgery) to have a good surgical outcome at the follow-up gait visit (good outcome rate of 71% vs. 33%). *Case* limbs were 1.2 times more likely than *control* limbs (i.e., limbs that had short gastrocnemius lengths but no lengthening surgery) to have a good outcome (71% vs. 59%). Three-fourths of the *case* limbs with a good outcome at the follow-up gait visit maintained this outcome over time, compared to only one-half of the *overtreated* limbs. Our results caution against over-prescription of gastrocnemius lengthening surgery and suggest gastrocnemius lengths can be used to identify good surgical candidates.

## Introduction

Equinus or “toe-walking” gait, characterized by excessive ankle plantarflexion in stance-phase of the gait cycle, is one of the most common gait patterns observed in patients with cerebral palsy (CP) [1]. This gait pattern is thought to arise from contracture and/or spasticity in one or more of the major plantarflexor muscles—the uniarticular soleus and biarticular gastrocnemius heads [2]. Clinical management of equinus gait includes conservative and surgical intervention to address these underlying mechanisms [2, 3]. Physical therapy, serial casting, and botulinum toxin type-A injections are often tried first to stretch and strengthen the muscles, improve ankle range of motion, and/or chemically denervate the muscles to dampen the effects of spasticity. If these approaches are unsuccessful, the gastrocnemius tendon is surgically lengthened, often as part of a single-event multi-level surgery (SEMLS) [4–6].

While gastrocnemius lengthening surgery is an effective intervention to improve gait for many patients with equinus, there is variability in patient response to surgery. Many patients exhibit improvements in static ankle range of motion [4, 7], ankle gait kinematics and kinetics [8], and other spatiotemporal parameters [9] at the post-operative clinical gait visit, and maintain these positive improvements into early adulthood [9, 10]. Unfortunately, others, either soon after surgery or in early adulthood, lose range of motion, develop crouch gait, or have recurrent equinus [4, 7, 9]. Previous work has identified several factors that influence likelihood of a good surgical outcome, including age at surgery and CP diagnosis subtype [4, 11]. However, it is unknown what other factors might differentiate patients who are likely to respond positively to surgery from those who are not.

We sought to understand the ability of model-based estimates of gastrocnemius muscle-tendon length (hereafter referred to as gastrocnemius length) during gait to predict short-term and long-term outcomes following a gastrocnemius lengthening surgery. Surgical recommendations are typically made using information collected at a clinical gait analysis, including kinematic, kinetic, electromyography, and physical exam data [12]. Previous work has shown how information derived from musculoskeletal modeling can complement these traditional datasets (e.g., [13–15]). Arnold and colleagues estimated peak muscle lengths during gait for the hamstrings using a computational musculoskeletal model that employed experimentally measured hip and knee kinematics [13]. This estimate of length, either on its own or as part of a statistical model, was an excellent predictor of improvements in crouch gait following a hamstrings lengthening surgery [13, 14], though less effective in predicting outcomes for those who required a repeat hamstrings lengthening surgery [15]. Similarly, we expect that a model-based estimate of peak gastrocnemius length in gait could measure if contracture of the gastrocnemius limits muscle length during walking and may indicate how likely a patient is to benefit from a gastrocnemius lengthening surgery.

We investigated two questions: (i) Do model-based estimates of gastrocnemius lengths differentiate which limbs are likely to receive a short-term benefit from a gastrocnemius lengthening surgery? and (ii) For the limbs that do achieve short-term benefits from surgery, are those benefits maintained long-term? We defined a good outcome from surgery to be an improvement in ankle kinematics with respect to the pre-surgical gait visit. These improvements were quantified using the Ankle Deviation Index, a metric we developed to capture the normalcy of ankle flexion kinematics during gait. We also created a gastrocnemius length “calculator” that can be used in a gait analysis clinic to help inform treatment decisions and improve long-term outcomes for patients undergoing a gastrocnemius lengthening surgery.

## Methods

### Data

We retrospectively analyzed the affected limb(s) of ambulatory patients with a diagnosis of CP seen at Gillette Children’s Specialty Healthcare Center for Gait and Motion Analysis. Institutional Review Boards (IRB) at Stanford University and Gillette Children’s Specialty Healthcare both approved this study. Patients, and guardians, where appropriate, gave informed written consent at the clinical visit for their data to be included in future studies. In accordance with IRB guidelines, all patient data was de-identified prior to any analysis.

**Fig 1:**
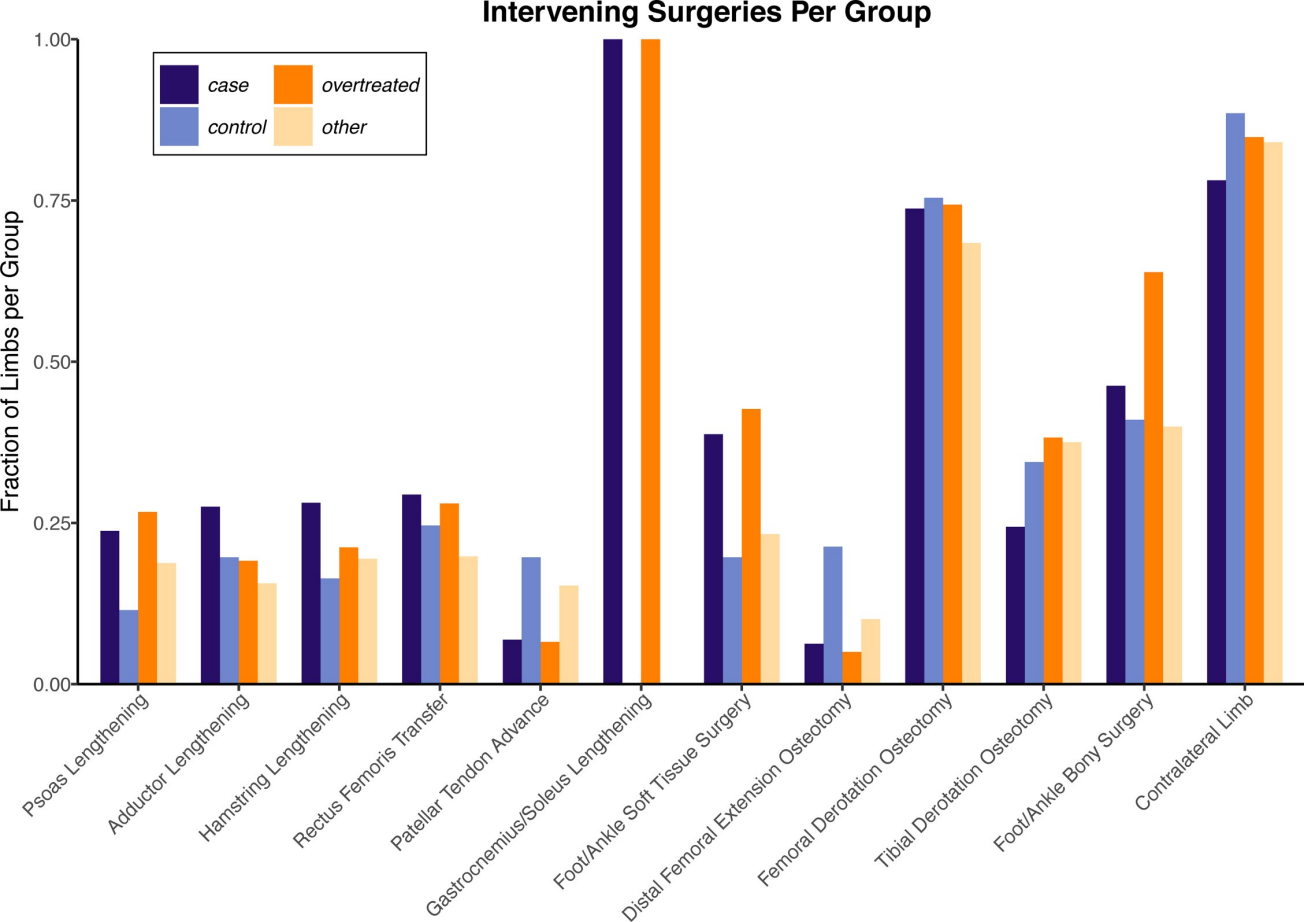
Intervening surgeries between analyzed gait visits. For the short-term analysis, each limb underwent at least two of the orthopedic procedures tabulated above (not including the “Contralateral Limb” count). Limb cohorts generally had similar intervening surgeries, though the *case* and *overtreated* limbs tended to have more concurrent surgical procedures at the foot and ankle level than the *control* and *other* limbs.

To be included in this study, the analyzed limb had to have at least one pair of gait visits in its clinical history with an intervening SEMLS (defined as having at least two orthopedic surgeries at a single surgical event). Exploratory or hardware removal surgeries were not considered to be therapeutic surgeries and did not contribute to this count (see Fig 1 for surgeries that met the inclusion criteria for a SEMLS). All treatment decisions were made based on recommendations from an interdisciplinary team of physicians, orthotists, and engineers upon review of gait analysis (i.e., kinematic, kinetic, and physical exam) data, medical history, and family and patient preferences. We split limbs into two groups. The “*+GL*” group were those limbs that underwent a gastrocnemius lengthening surgery at some point in their treatment history following their first gait visit (542 limbs from 398 patients). The “*- GL*” group were those limbs that did not undergo any gastrocnemius lengthening surgeries in between any pair of clinical gait visits (349 limbs from 252 patients). In total, we analyzed 891 limbs (650 individuals) over roughly 2,400 clinical visits. To our knowledge, this is the largest retrospective study to date examining outcomes from gastrocnemius lengthening surgery [9, 11].

For the short-term analysis, we examined one pair of consecutive pre- and post-surgery gait visits per limb. For the *+GL* limbs, we analyzed the pair of gait visits that surrounded the first surgical event that included a gastrocnemius lengthening procedure. For the *-GL* limbs, we chose a pair of gait visits that surrounded a surgical event that did not include a gastrocnemius lengthening. If a *-GL* limb underwent multiple orthopedic surgical events, we randomly chose one surgical event and the corresponding pre- and post-surgery gait visits for analysis.

### Computation of normalized gastrocnemius lengths in gait

**Fig 2:**
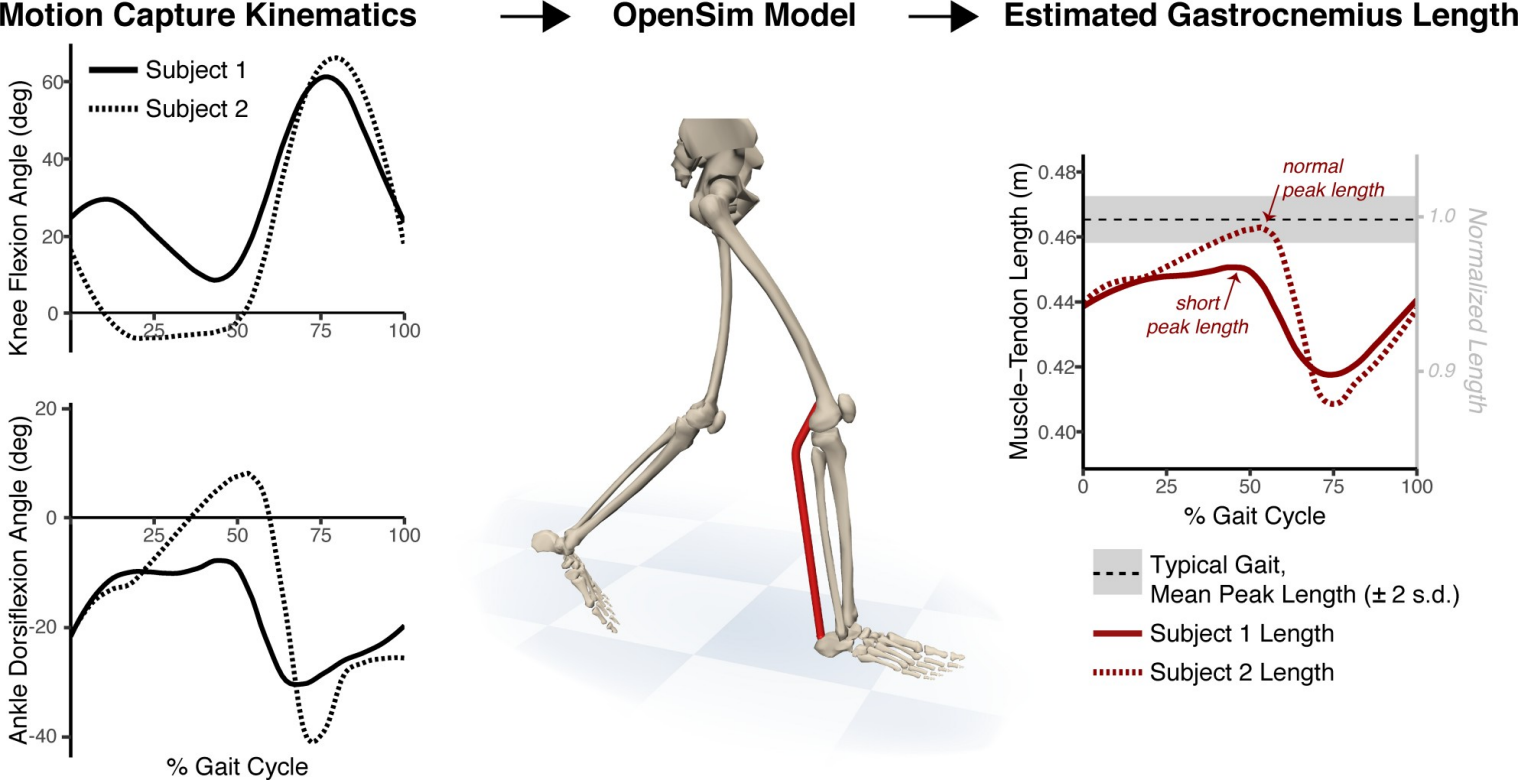
Method to estimate gastrocnemius length. Motion capture kinematics for knee flexion and ankle dorsiflexion were input to an unscaled OpenSim model [17, 18] to estimate gastrocnemius medialis muscle-tendon length over the gait cycle. These lengths were normalized to the average peak length computed for typical gait. Limbs whose peak length was at least 2 standard deviations below the typical mean (i.e., under the gray band in right the panel) were labeled as having a “short” gastrocnemius. Our supplemental “gastrocnemius length calculator” spreadsheet (S1 Worksheet) takes as input the motion capture kinematics pictured in the left panel and produces a gastrocnemius length graph as shown in the right panel. Two sample knee and ankle kinematic trajectories are shown. Subject 1 (solid lines) has increased knee flexion and ankle plantarflexion and a short peak gastrocnemius length. Subject 2 (dotted lines) has increased plantarflexion in early stance and throughout swing but attains normal peak gastrocnemius length in late stance.

Gastrocnemius lengths for each analyzed limb at the selected pre-surgery gait visit and for 147 typically developing limbs were estimated from ankle dorsiflexion and knee flexion kinematics and a computational musculoskeletal model (Fig 2). Kinematic data for the typically developing limbs were collected on healthy children (average age, 10.6 years) walking at self-selected speed [16]. These kinematic data were collected using a Vicon (Vicon, Oxford, UK) system and Plug-in-Gait marker set, and included three rotational degrees of freedom at the knee (flexion, adduction, and internal/external rotation) and ankle (flexion, inversion, and foot progression). The kinematic data collection protocol used for the typically developing and patient populations is described in detail by Schwartz et al. [16]. The ankle and knee flexion kinematics measured from the clinical gait analysis were normalized to the gait cycle and prescribed into a generic (i.e., unscaled) musculoskeletal model [17]. The model was implemented in OpenSim [18], an open-source musculoskeletal simulation software, and characterized the bony geometry, joint axes, and muscle attachments of the lower extremity. The model included a 1 degree-of-freedom knee joint (flexion/extension) with translational and non-sagittal rotational degrees-of-freedom coupled to the knee flexion angle, and a 1 degree-of-freedom revolute joint at the ankle (dorsiflexion). Non-sagittal ankle degrees-of-freedom in the model were locked for this study. Details of the musculoskeletal model are described by Rajagopal et al. [17]. OpenSim was used to compute the model’s gastrocnemius medialis muscle-tendon length over the gait cycle from the specified kinematics.

From these computations, we extracted the peak muscle-tendon length per observation over the gait cycle. For the set of typically developing limbs, we computed the mean (*μ_TD_*) and variance ($\sigma_{TD}^{2}$) of these peak muscle-tendon lengths. CP limbs were labeled as having a “short” gastrocnemius if the peak muscle-tendon length for that limb (*l_CP_*) was at least two standard deviations below the typical gait peak length (i.e., *l_CP_*≤*μ_TD_*−2*σ_TD_*). We said a limb “met the criterion” for a gastrocnemius lengthening surgery if the limb was labeled as having a short gastrocnemius.

### Definition of limb treatment groups

All analyzed limbs were cross-classified based on if the limb met our defined criterion for surgery and if the limb underwent a gastrocnemius lengthening surgery. This resulted in four treatment groups, named based on our hypothesized surgical outcomes: *case* limbs (*n* = 160), that had a short gastrocnemius and underwent gastrocnemius lengthening surgery; *control* limbs (*n* = 61), that had a short gastrocnemius but did not undergo gastrocnemius lengthening surgery; *overtreated* limbs (*n* = 288), that did not have a short gastrocnemius but underwent gastrocnemius lengthening surgery; and *other* limbs (*n* = 382) that did not have a short gastrocnemius and did not undergo gastrocnemius lengthening surgery. We hypothesized *case* limbs would have better surgical outcomes than the *control* limbs whose peak pre-operative gastrocnemius lengths suggested good candidacy for a gastrocnemius lengthening surgery. We also hypothesized *case* limbs would have better outcomes than the *overtreated* limbs whose peak pre-operative gastrocnemius lengths did not indicate a need for a gastrocnemius lengthening surgery.

Summary statistics about limbs in the four groups are included with the supplementary materials (S1 Data). Notably, the ages at the pre-surgical visit were similar between the four groups; the mean (standard deviation) ages, in years, for the *case*, *control*, *overtreated*, and *other* limbs were 9.1 (3.3), 10.1 (3.5), 9.8 (3.1), and 10.5 (3.2), respectively. The mean (standard deviation) elapsed time between the pre-surgical and post-surgical gait visits was 1.6 (0.6) years and mean elapsed time between the surgery and post-surgical gait visits was 1.2 (0.5) years. Other than gastrocnemius lengthening, limbs in each of these four groups underwent similar orthopedic surgical procedures between the pair of analyzed gait visits, though the *case* and *overtreated* limbs tended to have more concomitant soft tissue and bony foot- and ankle-level surgeries (Fig 1).

### Outcome analysis

**Fig 3:**
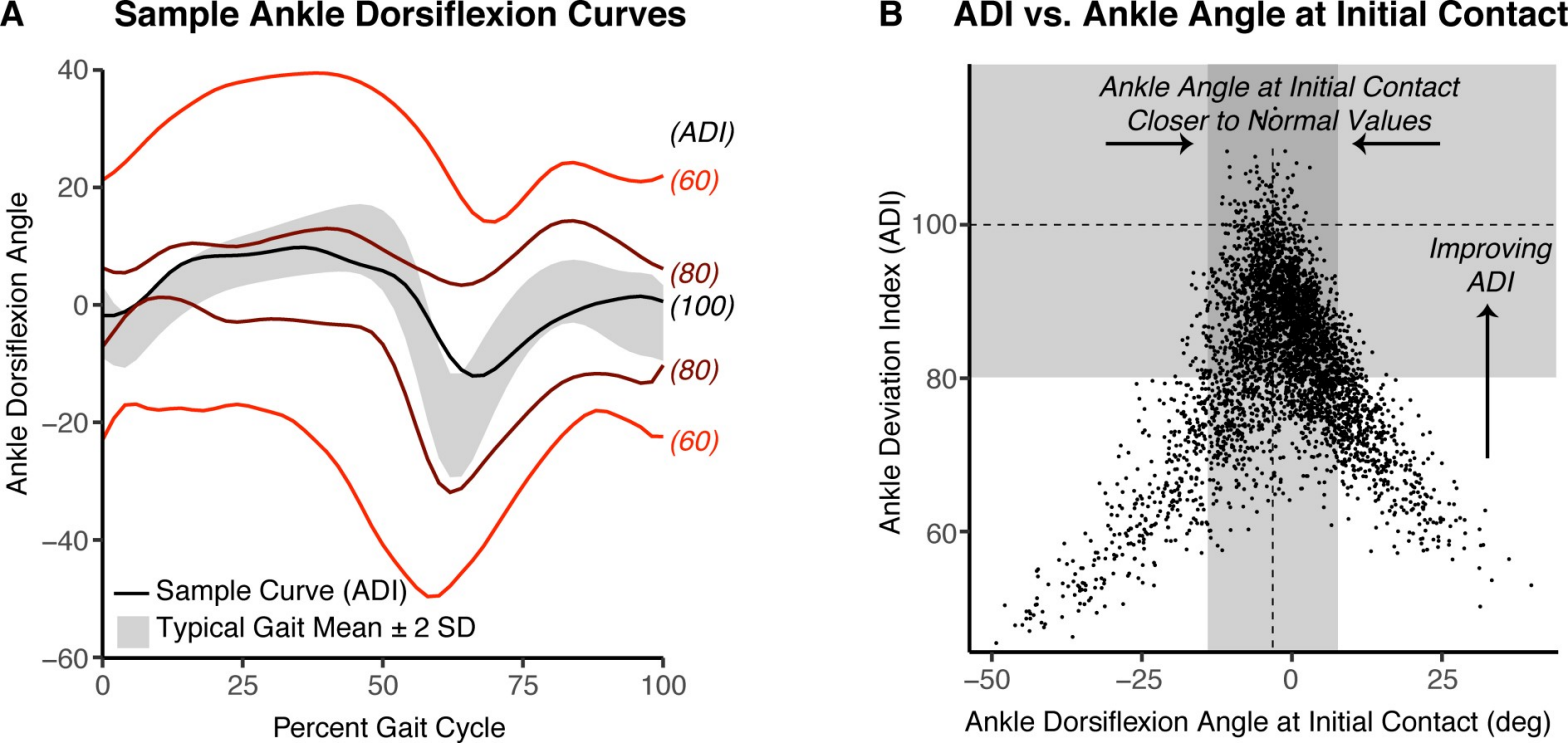
Ankle Deviation Index as indicator of ankle kinematics normalcy. The Ankle Deviation Index (ADI) is a summary metric that quantifies the normalcy of the ankle dorsiflexion angle time series with respect to typical gait. Typical gait has a mean ADI of 100, with every 10 point decrement representing one standard deviation away from the mean. (A) Sample kinematic trajectories and the corresponding ADI are shown. (B) Improvement in ADI is associated with improvements in key biomechanical parameters of gait, including ankle dorsiflexion angle at initial contact (pictured), mean stance dorsiflexion angle, peak dorsiflexion angle in swing, and others. Each point represents an observation used in the development of the ADI.

As gastrocnemius lengthening surgery is commonly prescribed as an intervention to improve ankle kinematics, we defined an ankle-specific outcome metric—the Ankle Deviation Index (ADI)—that measures the normalcy of the ankle flexion kinematics time series with respect to typical gait (Fig 3). Computation of the ADI followed a method similar to the Gait Deviation Index [19], but used only the ankle flexion kinematics time series. For reference, in typical gait, ADI ~ $N(\mu =100,\sigma^{2}=100)$, and every 10-point decrement from 100 represents 1 standard deviation away from typical gait. Sample kinematic time-series curves and their associated ADI are provided for the reader to develop an intuition for the mapping between the original kinematic data and the computed ADI (Fig 3A). We verified that as ADI increases, key biomechanical parameters of gait, e.g., dorsiflexion angle at initial foot contact (Fig 3B), peak dorsiflexion angle in stance and swing, and others, are closer to their respective normative values. Thus, an improvement in ADI can simultaneously capture improvements in the multiple key ankle kinematic metrics that are often targets of gastrocnemius lengthening surgery.

For this study, we labeled a post-surgery gait visit as having a “good” outcome if there was either at least a 5-point improvement in ADI compared to the pre-surgery gait visit (i.e., one-half standard deviation closer to normal post-surgery compared to pre-surgery), or any improvement in ADI from the pre-surgery gait visit, with a final ADI of above 90 (i.e., within 1 standard deviation of typical gait mean ADI). All other outcomes were labeled as “poor”. This definition was based on definitions of good outcomes using other similar gait metrics [20, 21]. With this definition, a good outcome label indicated ankle kinematics were strictly closer to normal post-surgery compared to pre-surgery. For example, a limb in excessive plantarflexion throughout gait that had a pre-surgery ADI of 75 and post-surgery ADI of 80 would be labeled as good, regardless if the post-surgery ankle kinematics were on the dorsiflexed or plantarflexed side of normal.

To estimate the efficacy of the gastrocnemius peak lengths as a predictor of short-term improvement in ankle kinematics following gastrocnemius lengthening surgery, we computed the fraction of limbs in each of the *case*, *control*, *overtreated*, and *other* groups that were labeled as good outcomes at the post-surgical visit. We used Pearson’s *χ*^2^ analysis to test for association between our defined surgical selection criterion (short vs. not short gastrocnemius) and post-surgery good outcome rate. In each group, we also computed change from the pre- to post-surgical values of ADI, mean stance dorsiflexion, and mean stance knee flexion to test for magnitude of improvement from surgery and risk for devolvement into crouch gait for over-treated individuals. We used the Tukey method [22] to test for significant differences in the pre- to post-surgical change between the *case* limbs and *control*, *overtreated*, and *other* limbs.

**Fig 4:**
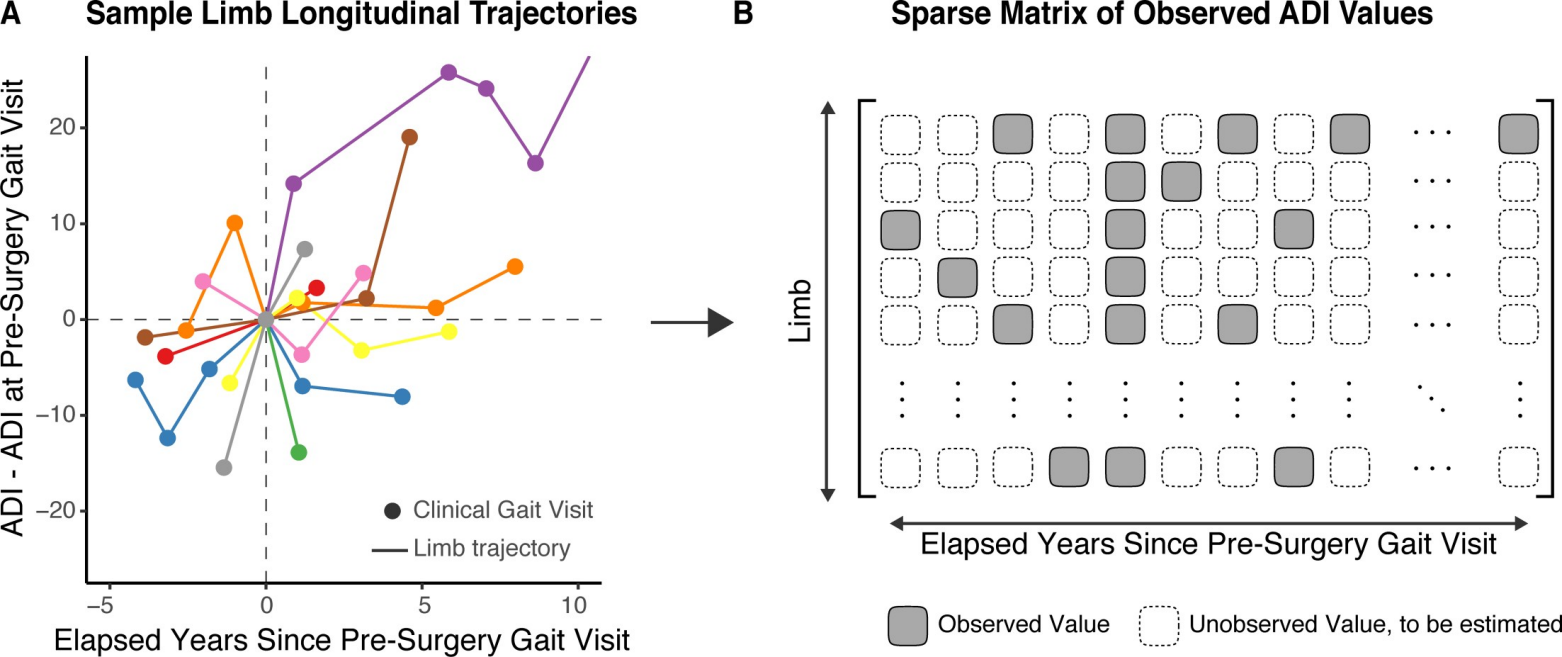
Longitudinal data for long-term analysis. Patients are seen for a gait analysis multiple times throughout their clinical history. (A) The number and frequency of these visits are variable per limb but serve as valuable sparse observations of a limb’s longitudinal development following orthopedic surgery. (B) These observed data can be stored in a sparse matrix. Matrix completion algorithms [23] can be used to estimate the unobserved values by borrowing strength from the available observed values and compute a robust mean patient trajectory following surgical treatment.

In the *case* and *overtreated* limbs, we characterized the ability of pre-surgery gastrocnemius peak length to track the long-term maintenance of benefits from gastrocnemius lengthening surgery. Because we analyzed a retrospective, observational dataset, we could not specify the time nor frequency with which a limb is called back to the clinic for re-evaluation. Consequently, each limb has a variable number of irregularly spaced observations at the clinic (illustrative examples with variable quantity and temporal spacing of gait visits shown in Fig 4A). To address our question about long-term maintenance, we took two separate approaches: First, for each of the *case* and *overtreated* groups, we binned observations in our dataset based on the number of years elapsed since the gastrocnemius surgery and computed the fraction of limbs in each group and time bin labeled as a “good outcome”. To examine if observed good outcomes at the initial post-surgical gait visit were maintained at repeat visits, we repeated this analysis after further cross-classifying limbs based on the post-surgical gait visit outcome. This method provides an estimate of conditional good outcome rate in each group as discrete function of the time bin queried. In other words, “how likely is a good outcome over time, given a good outcome initially?”. Finally, in the *case* and *overtreated* groups, we used a sparse-longitudinal matrix completion [23] to estimate the mean progression of the ADI as a function of years elapsed from the pre-surgery gait visit. Briefly, this method organizes the infrequently and irregularly observed data into a sparse matrix (Fig 4B) and imputes the unobserved data using low-dimensional matrix factorization. Once the missing observations are imputed, the group’s average trajectory can be estimated as the mean of the imputed individual limb trajectories. In our case, even though we only have, on average, 1.9 and 2.5 post-surgery gait visits per *case* and *overtreated* limb, respectively, the irregular spacing of these visits works to our advantage as we can leverage information from the available observations to impute the missing observations. To estimate the confidence bounds for both the *case* and *overtreated* group means, we ran 200 bootstraps, with a random 75% of the respective limbs in each run. We used the Hotelling *T*^2^ test [24] with the mean curves sampled each year after the initial gait visit to test if these curves were statistically different.

### Gastrocnemius length calculator spreadsheet

We created an easy-to-use workbook to aid gastrocnemius length calculation from clinical gait analysis data (S1 Worksheet). To generate a gastrocnemius length plot (e.g., as in Fig 2, right panel), users simply enter the experimentally measured knee and ankle flexion kinematics (e.g., the data visualized in Fig 2, left panel) into the main spreadsheet. Gastrocnemius lengths are automatically plotted from these entered kinematics using a reference table of gastrocnemius lengths as a function of knee flexion and ankle dorsiflexion angle that is included with the workbook. The workbook also includes reference mean and standard deviation of typical gait gastrocnemius lengths over the gait cycle.

## Results

**Fig 5:**
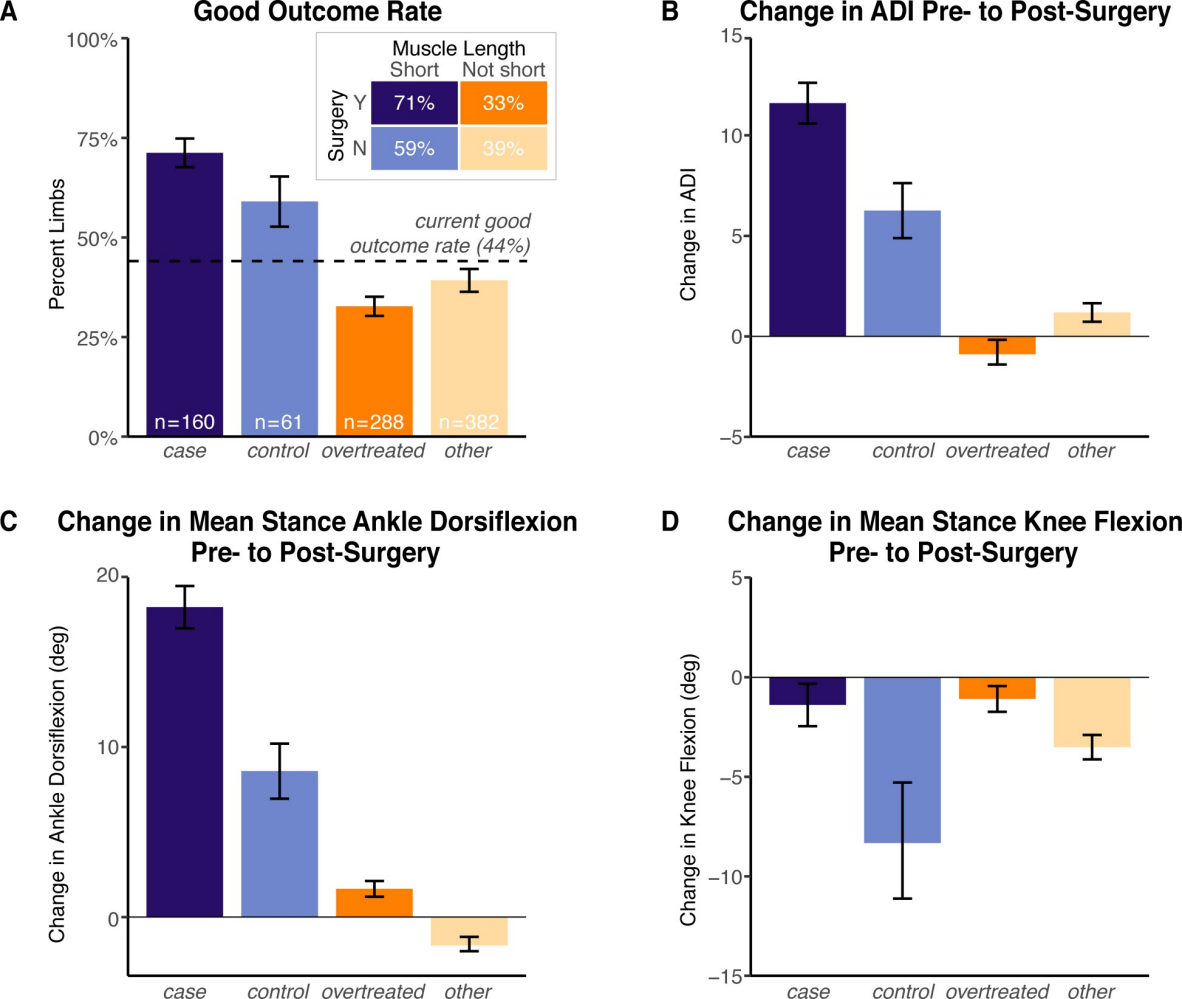
Cross-classified short-term improvements following gastrocnemius lengthening surgery. Limbs were cross-classified based on those that underwent a gastrocnemius lengthening surgery (*case* and *overtreated*) and those labeled as having a short pre-surgery gastrocnemius (*case* and *control*). (A) Good outcome rates, defined as the fraction of limbs that had at least a 5-point improvement in ADI, or an improvement in ADI with post-surgery ADI > 90, were computed per group. The current good outcome rate was computed from retrospective data from all limbs that underwent gastrocnemius lengthening surgery. Changes in the (B) ADI, (C) mean stance ankle dorsiflexion angle, and (D) mean stance knee flexion angle were computed from the pre-surgery to post-surgery clinical gait visit. Kinematic improvements in the *case* limbs exceeded those of the *overtreated* and *control* limbs. Limbs, on average, did not tend to devolve into a crouch gait, even within the *overtreated* limbs. In all panels, error bars represent ± 1 standard error of the mean.

The *case*-*control*-*overtreated*-*other* classification was able to distinguish likelihood of a good short-term (i.e., at the post-surgery gait visit) outcome (*χ*^2^ = 66, *p* = 3.8e-16). The good outcome rate in *case* limbs, 71% (standard error = 3.6%), exceeded that in the *overtreated* limbs, 33% (standard error = 2.4%), the *control* limbs, 59% (standard error = 6.3%), and the historical good outcome rate, 44%, estimated from retrospective data (Fig 5A). The mean ADI improvement from pre- to post-surgery in the *case* limbs was 11.6 points, which was greater than the mean 0.9-point drop in the *overtreated* limbs (*p* = 9.3e-14) and the 6.3-point improvement in the *control* limbs (*p* = 0.004). *Other* limbs had a 1.2-point improvement in ADI from the pre- to post-surgery visit (Fig 5B). These ADI changes were consistent with improvement in other measures of ankle kinematics, such as mean stance ankle dorsiflexion (Fig 5C). For the limbs that underwent a gastrocnemius lengthening surgery (*case* and *overtreated*), changes in mean stance knee flexion from pre- to post-surgery were not significantly different, and not significantly different from 0 (Fig 5D).

**Fig 6:**
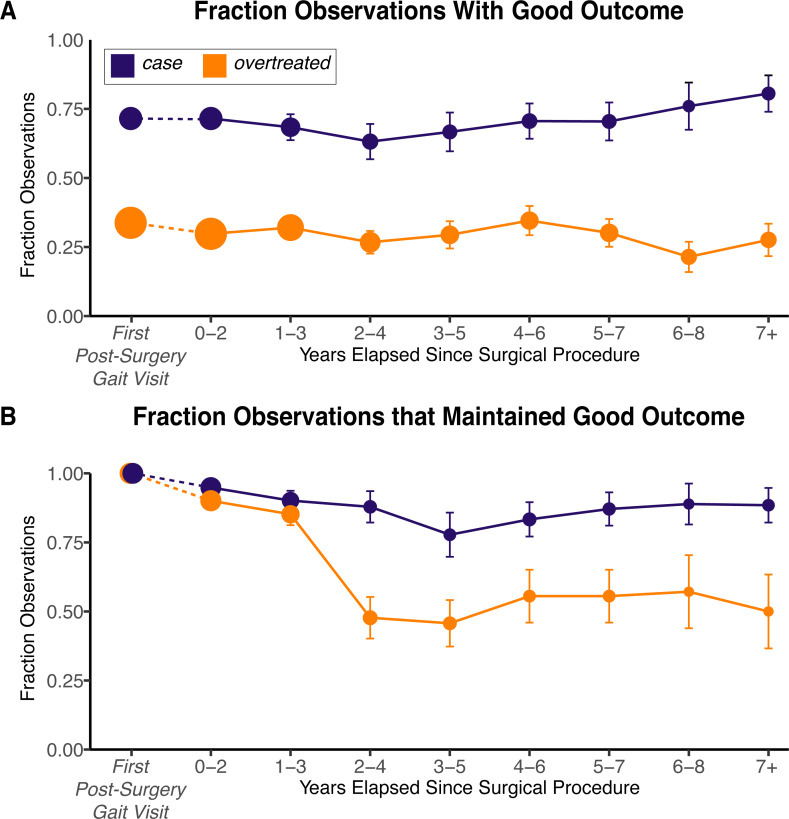
Long-term outcomes following gastrocnemius lengthening surgery in *case* and *overtreated* limbs. Observations were labeled as good outcomes if either the improvement in ADI with respect to the pre-surgery gait visit was at least five points, or if the ADI was greater than the pre-surgery visit ADI and at least 90. (A) These observations were binned based on years elapsed following the gastrocnemius lengthening procedure, and rate of good outcome in each bin was computed. (B) Observations were filtered based on whether the limb was labeled as a good outcome at the post-surgical gait visit to examine if the observed outcomes immediately following surgery were maintained over time. The error bars represent ± 1 standard error for the computed fraction. The areas of the dots in (A) and (B) are proportional to the number of observations included in that bin.

**Fig 7:**
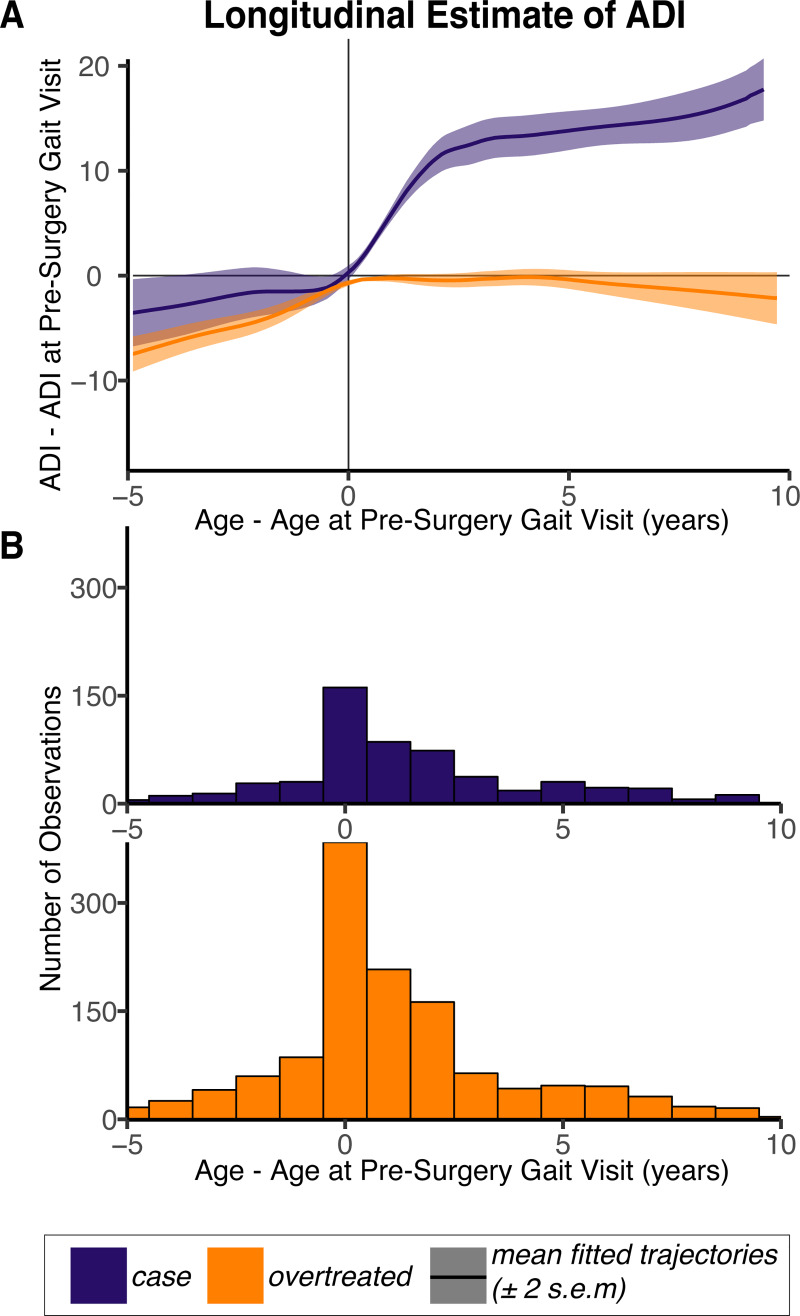
Longitudinal estimates of ADI in *case* and *overtreated* limbs. (A) The mean ADI improvement following surgery was estimated as a function of time elapsed since the pre-surgery gait visit. *Case* limbs had a greater improvement in ADI than the *overtreated* limbs at equivalent times. The shaded bands represent the 95% confidence interval for the *case* and *overtreated* group mean trajectories. (B) These longitudinal *case* and *overtreated* group estimates were based on available observations, which decrease in number as time from surgery increases.

When examining long-term outcomes of the gastrocnemius lengthening surgery (i.e., time points *after* the first post-surgery visit), the fraction of *case* and *overtreated* limbs that were labeled as “good” relative to the pre-surgery visit remained roughly constant (Fig 6A). When limiting this analysis to only those limbs that had a good outcome at the first post-surgery visit, we found that over three-fourths of the *case* limbs maintained their good short-term outcome over the long-term, compared to only half of the *overtreated* limbs (Fig 6B). The mean longitudinal trajectories for the *case* and *overtreated* limbs indicate that the observed short-term changes in ADI (i.e., the 11.6-point improvement in the *case* limbs and the 0.9-point loss in the *overtreated* limbs) are maintained over time; and the average improvement in ADI for the *case* limbs was higher than the average improvement in ADI for the *overtreated* limbs at equivalent times (Fig 7A). Uncertainty in these trajectory estimates increase with elapsed time as the number of available observations decreases (Fig 7B).

## Discussion

We have defined a surgical selection criterion for gastrocnemius lengthening surgery based on whether the peak gastrocnemius length in gait was “short” (i.e., at least 2 standard deviations below the mean peak length reached in typical gait). Of the limbs that underwent a gastrocnemius lengthening surgery, the *case* limbs that met our criterion were 2.2 times more likely to have a good short-term outcome than the *overtreated* limbs that failed our criterion (71% vs. 33%) (Fig 5). Of the limbs that met our surgical criterion, the *case* limbs that had a gastrocnemius lengthening surgery were 1.2 times more likely to have a good short-term outcome than the matched *control* limbs that did not have a gastrocnemius lengthening surgery (71% vs. 59%). The *case* limbs were also far more likely than the *overtreated* limbs to maintain a good outcome observed at the post-surgery gait visit (Fig 6B). These results caution against over-prescribing a gastrocnemius lengthening surgery, and suggest that estimating gastrocnemius lengths during gait could be a valuable evaluation tool when considering whether a patient should undergo a gastrocnemius lengthening surgery. We provide an easy-to-use spreadsheet to generate these muscle length estimates from ankle flexion and knee flexion kinematics (S1 Worksheet).

While the average improvements in kinematic variables among all limbs that underwent a gastrocnemius lengthening surgery in our dataset were similar to those reported in the literature (e.g., [7, 9, 11, 25, 26]), our simple surgical selection criterion was able to separate out the limbs that achieved significantly larger improvements. For example, Dreher and colleagues conducted a longitudinal study of 82 limbs that underwent a gastrocnemius lengthening procedure and found that mean stance ankle dorsiflexion angle improved by 9° from pre- to post-surgery [9]. This average improvement is comparable to the 7° improvement in our dataset among all limbs that underwent a gastrocnemius lengthening surgery. However, the magnitude of improvement was not constant between the *case* and *overtreated* limbs: in our study, the *case* limbs improved by, on average, 18° from pre- to post-surgery, while the *overtreated* limbs improved by only 2° (Fig 5C). This pattern of improvement (i.e., the larger observed improvements in the *case* limbs compared to the *overtreated* limbs) was consistent among other key ankle kinematic metrics (e.g., dorsiflexion angle at initial contact and peak dorsiflexion angle in stance) and in the ADI (Fig 5B).

Our selection criterion also differentiated the limbs that were likely to have long-term benefits from gastrocnemius lengthening surgery. When estimating likelihood of a good long-term outcome using only information from the pre-surgery gait visit (i.e., gastrocnemius length), we found that *case* limbs had at least a two-thirds likelihood of being labeled as “good” relative to the pre-surgery visit, while *overtreated* limbs had at most a one-third likelihood (Fig 6A). Further conditioning the long-term analysis on the observed short-term outcomes gives more information about how likely it is for a good post-surgery outcome to be maintained in the long-term. Of the *case* limbs that had a good short-term outcome, over three-fourths maintained their good outcome in the long-term, while only half of the *overtreated* limbs with a good short-term outcome maintained their good outcome in the long-term (Fig 6B). This suggests that any short-term improvements observed in the *case* limbs are more likely to be maintained over the long-term than improvements observed in the *overtreated* limbs. These results are consistent with our estimates of mean improvement in ADI in the *case* and *overtreated* limbs as a function of time elapsed since the pre-surgical gait visit (Fig 7A).

Given these findings, a possible interpretation of our estimated normalized gastrocnemius lengths is as a kinematically-derived functional measure of the severity of a limb’s plantarflexor contracture. As contracture severity, and therefore passive resistance to muscle stretch, increases, the expected peak attained length of the gastrocnemius in gait decreases. In this study, our musculoskeletal model served as a biomechanical transfer function between experimentally measured ankle and knee flexion kinematics and lengths of the biarticular gastrocnemius.

Even with simplifications in our musculoskeletal modeling pipeline, our estimated gastrocnemius lengths were able to define meaningful surgical criteria that could improve current rates of success from surgery. The musculoskeletal model used in this study did not capture any effects of patient-specific musculoskeletal geometry on muscle length. Our analysis did not exclude any patients based on abnormal clinical measurements of skeletal geometry (e.g., excessive tibial torsion), nor did we exclude patients based on prior osteotomies that may have altered their skeletal geometry. Moreover, the motion capture data used in this study treated the foot as a rigid segment and did not capture effects of mid-foot breakdown on measured ankle flexion angle [27]. Future work that incorporates these patient-specific variations in musculoskeletal geometry, such as using patient-specific models built from imaging data (e.g., [28, 29]), along with more nuanced measurements of foot-segment kinematics may be able to derive more discriminatory power from calculated gastrocnemius lengths on surgical outcomes.

It is important to note that, even with the incorporation of patient-specific geometry, the normalized gastrocnemius length is a geometric calculation, and does not provide any information about the muscle fiber length, force, or force-generating capacity. To capture this information, we would need to incorporate patient-specific measurements of muscle architecture including optimal fiber length, muscle volume, tendon slack length, muscle and tendon passive stiffness, and muscle activation levels. Encouragingly, researchers have shown how modeling the effects of elements such as muscle contracture, weakness, or spasticity within a simulation pipeline may reproduce some experimental observations in muscle activity and gait (e.g., [30–32]). However, muscle architecture parameters and neural control vary widely among individuals with cerebral palsy [33, 34], and we do not currently have a means for accurately measuring them.

Some limitations of our study should be noted. First, we only analyzed data from a single center. Other clinical centers likely have differing treatment philosophies, including ages when surgery is prescribed, frequency of single-level vs. multi-level surgery, and variations in surgical technique. For example, in our dataset, most gastrocnemius lengthening surgeries were performed using the Strayer procedure [35]. Historically, this procedure has been reported to have relatively low rates of poor outcomes [11], and likelihood of adverse outcomes may differ with different surgical techniques. We suggest centers retrospectively validate our predictions on their own data before adopting our model for prospective use, and we encourage other centers to share conclusions learned from analyzing their own data.

Second, the knee and ankle kinematic degrees-of-freedom differed between the Plug-in-Gait model used as part of the gait analysis protocol to compute joint kinematics [16] and the OpenSim musculoskeletal model [17] used to compute gastrocnemius lengths from these kinematics. Previous work has shown that differences in the underlying skeletal model used to compute joint kinematics can impact knee and ankle flexion angle estimates by an average of 2° to 6° [36], and these differences in joint angles may have impacted our estimated gastrocnemius lengths. Future work that characterizes the impact of this error on the predictive power of muscle lengths on surgical outcomes would be valuable.

Third, deviations in ankle kinematics may be a consequence of more than just gastrocnemius contracture, and improvement in ankle kinematics likely depends on appropriate surgical correction of concomitant musculoskeletal abnormalities [37]. For our dataset 42% of all gastrocnemius lengthening procedures were performed with accompanying soft tissue surgery around the foot and ankle, and 59% with accompanying bony surgery around the foot and ankle (Fig 1). The confounding effect of these simultaneous surgical procedures on short-term and long-term outcomes was not analyzed in this study but is important to consider when interpreting our results.

Fourth, much of our analysis focused on the diverging outcomes of the *case* and *overtreated* limbs, but the outcomes of the *control* limbs need to be further examined. The *control* limbs were identified to have short gastrocnemii, but, unlike the *case* limbs, did not undergo a gastrocnemius lengthening surgery. While the rate of short-term good outcomes (59%) and average short-term ADI improvement (6.3) in the *control* limbs was lower than in the *case* limbs (71% and 11.6, respectively), many of the *control* limbs had improvements in ankle kinematics following surgery (Fig 5). It is unclear what treatment and patient-intrinsic effects in the *control* limbs are driving these improvements, but it is likely the multi-level surgeries have coupled, beneficial effects at the ankle that merit further investigation. Natural progression, especially at younger ages [38], could also affect the perceived benefits of surgery, but we expect these effects are similar between the groups analyzed since the average ages at the pre-surgery gait visit were similar. Our dataset contained too few *control* observations to do an effective, long-term analysis tracking how these *control* limbs evolved in relation to the *case* limbs. Future work to address these open questions on short-term and long-term effectiveness of multiple approaches to improve ankle kinematics would be valuable.

Fifth, we analyzed the marginal effectiveness of a gastrocnemius lengthening surgery as part of a SEMLS but did not analyze the effectiveness of surgical intervention to improve gait compared to no surgical intervention. The natural progression of gait with age in the cerebral palsy population is variable—after the initial gait maturation through approximately the first seven years of life [38], gait function stabilizes with age for some patients [38, 39] but declines with age for others [40, 41]. Similarly, studies examining the effect of surgery relative to natural progression have reported that surgery is an effective intervention for improving gait that can offset expected gait decline for some patients [42] but may provide limited clinically meaningful improvements relative to natural progression for other patients [21]. Results from our study should be considered in parallel with these other findings to build a more robust estimate of the potential effectiveness of surgery.

Finally, we analyzed a relatively simple measure of outcome—improvement in the ADI relative to the pre-surgery gait visit. This outcome metric was designed to specifically track normalcy in ankle kinematics. While more normal ankle kinematics are associated with better stance foot stability, better swing foot clearance, and healthier loading patterns on the foot [43], they are not direct measures of functional capacity. Future work that examines the predictive capacity of the surgical criteria we defined in our study on functional outcomes would be valuable.

## Conclusion

We have built and shared a simple tool to inform clinical decisions about the inclusion of a gastrocnemius lengthening surgery to improve ankle kinematics. Our findings suggest that limbs with a short gastrocnemius in gait (*case* limbs), as estimated by our model, are more likely than the limbs without a short gastrocnemius (*overtreated* limbs) to have and maintain meaningful improvements in ankle kinematics over time following a gastrocnemius lengthening surgery. Our retrospective *case*-*control* analysis suggests that some of the ankle kinematic improvements seen in the *case* limbs are likely due to other concomitant surgeries, and future work to identify the effectiveness of multiple approaches to improve ankle kinematics would be valuable. We encourage clinical centers to test these findings and share their results.

## Supporting information

S1 Data Kinematic data analyzed in this study. This file contains two Microsoft Excel spreadsheets: (1) “data”, which contains longitudinal data on limb age, elapsed time since pre-surgery gait visit and since surgery, ADI, mean stance ankle dorsiflexion, mean stance knee flexion, peak gastrocnemius length, class (i.e., *case*/*control*/*overtreated*/*other*), and outcome label (i.e., good/bad); and (2) “summary-stats”, which contains summary data describing patient demographics, clinical severity, and surgical treatment.
(XLSX)

S1 Worksheet Clinical worksheet to estimate gastrocnemius lengths from gait analysis data. This file contains four Microsoft Excel sheets: (1) “Surgery Calculator”, where users enter knee and ankle flexion kinematics normalized to the gait cycle; a plot of gastrocnemius length over the gait cycle is generated from these input data; (2) “GasMed_Lengths_REF”, which contains a reference table of gastrocnemius lengths computed from the musculoskeletal model used in this study; (3) “GasMed_Lengths_TD”, which contains reference data for the mean and standard deviation of gastrocnemius length over the gait cycle for typical gait; and (4) “Summary Statistics”, which contains summary data describing patient demographics, clinical severity, and surgical treatment.
(XLSX)
